# Editorial: Chronic orofacial pain

**DOI:** 10.3389/fpain.2022.1086256

**Published:** 2023-01-04

**Authors:** Caroline Machado Kopruszinski, Frank Porreca, Juliana Geremias Chichorro

**Affiliations:** ^1^ Department of Pharmacology, College of Medicine, University of Arizona, Tucson, AZ, United States; ^2^ Department of Neurology, Mayo Clinic, Phoenix, AZ, United States; ^3^ Department of Pharmacology, Biological Sciences Sector, Federal University of Parana, Curitiba, Brazil

**Keywords:** trigeminal nerve, pharmacological treatment, orofacial pain mechanisms, temporomandibular disorders – TMD, burning mouth syndrome (BMS), migraine

**Editorial on the Research Topic** Chronic orofacial pain

Chronic orofacial pain includes multiple pain conditions of musculoskeletal, neurovascular, nociplastic and neuropathic origin and is characterized as pain affecting the oral, head, face, and neck area for more than 15 days per month and lasting for more than 4 h daily for at least the past 3 months (1, 2). These conditions usually represent a clinical challenge, both in terms of diagnosis and treatment, due to diversity, complexity and lack of deep knowledge about etiology and pathogenesis which seem to be relatively different from those disorders involving spinal nerves. Temporomandibular disorders (TMDs) consist of a heterogeneous family of musculoskeletal disorders that represent the most common chronic orofacial pain condition. In contrast, trigeminal neuralgia is a relatively rare but extremely severe form of neuropathic pain. Moreover, some chronic orofacial pain conditions, such as burning mouth syndrome (BMS), are multifactorial and poorly understood, and may be very frustrating for the clinician and the patient. Treatment options for chronic orofacial pain are limited and management is further complicated by the frequent co-expression of one or more comorbidities such as depression, anxiety, stress, sleep disturbances and widespread sensory abnormalities (1–4). Most chronic orofacial pain conditions have a higher female prevalence, suggesting sex differences in trigeminal pain development and chronification (2–4). This research topic includes five clinical and preclinical articles on humans and rodents, which contribute to the current understanding of TMD, migraine and BMS with implications for diagnosis and development of novel therapeutic strategies.

TMD comprises complex biological mechanisms, such as activation of the immune system, activation of the inflammatory process, and degradation of extracellular matrix (ECM) components that lead to pain and/or dysfunction in the temporomandibular joints, masticatory muscles, and associated regions (5). A better understanding of the pathophysiology of TMD might contribute to an early diagnosis, management and possibly a decrease in the frequency of the pain and potential chronification. Ferreira et al. reviewed the action of the most studied ECM component, hyaluronic acid (HA) (6), as a damage-associated molecular pattern (DAMPs) on the etiopathogenesis of TMD. High-molecular-weight HA presents anti-inflammatory properties in healthy tissues, however, low-molecular-weight HA as a product of high-molecular-weight HA catabolism after a tissue injury is proinflammatory and promotes the activation and maturation of immune cells and the release of pro-inflammatory cytokines. The authors highlighted in the review that low-molecular-weight HA activity as DAMP may play a significant role in inflammatory TDM. Moreover, orofacial chronic pain has been linked with several differences in brain structure, function, and neurochemistry as well as in the processing of sensory stimuli. Smith et al. recently revealed in this research topic the first pilot study of graph theoretical approaches to resting state data in TMD patients. The group applied resting-state functional MRI and task-based functional MRI to compare changes in the trigemino-thalamocortical, the sensory/discriminatory lateral and affective/cognitive medial pain systems, motor system, and default mode network in patients with TMD in comparison with other chronic pain disorders and pain-free individuals. The authors suggested that TMD disorders may be associated with an increase in functional connectivity in resting or evoked-pain conditions, however, additional studies are required to further characterize these changes, replicate these results, increase the number of participants, incorporate structural connectivity as well as establish causal associations among the network nodes delineated. In addition, approximately 10% of TMD individuals present the transition from acute to chronic pain states in which pain intensity has been suggested as a significant contributing factor (5, 7). Velly et al. reported in the current research topic a 3-month prospective cohort study involving 109 TMD patients that were evaluated regarding pain intensity, pain always being present, pain or stiffness on awakening, jaw activities, and interference. The authors conclude that characteristic pain index, moderate to severe average pain intensity, pain always present, and interferences were associated with the transition from acute to chronic pain when compared at the first and third month of follow-up visits. These factors might be further taken into consideration when evaluating and developing treatment strategies for patients with pain associated with TMD.

Temporomandibular joint disorders are conditions often comorbid with migraine. In a previous study, it was demonstrated that dietary inclusion of a grape seed extract (GSE) was shown to inhibit trigeminal pain signaling *via* involvement of 5-HT3, 5-HT7, and GABAB receptors in rats subject to neck muscle inflammation animals (8). In the article included in this research topic, Woodman et al. reported the effect of daily supplementation with GSE in migraine model, that consists of promoting latent sensitization of trigeminal ganglion neurons *via* restraint stress prior to exposure to the pungent compound umbellulone (9). GSE caused a significant reduction of facial and hindpaw mechanical allodynia induced by exposure to pungent odors, an effect mediated by cannabinoid CB1 and CB2 receptors. In addition, GSE reduced CGRP expression in the spinal trigeminal nucleus, induced by the odor's exposure. Interestingly, the authors did not observe sex differences in the intensity of the nociceptive responses nor the effect of GSE, however they have used adolescent rats. It is now widely accepted that after puberty migraine incidence increases at a much higher rate in females than males and over the life course occurs in women three to four times more often than in men (10). Thus, it raises another interesting question: would the effect be the same if GSE was tested in adult male and female rats? In spite of that, Woodman and colleagues showed a clear benefit of GSE supplementation that encourages further preclinical and clinical studies in orofacial pain conditions.

Lastly, Watanabe et al. performed a retrospective study comparing the effectiveness of amitriptyline and aripiprazole in the management of BMS in elderly patients. They reported superior efficacy of amitriptyline compared to aripiprazole (around 54% and 41%, respectively). Additionally, amitriptyline was better tolerated, is spite of the high incidence of anticholinergic effects, such as constipation, dizziness, dry mouth, and drowsiness. The main side effect related to aripiprazole was sleep disorder. It is noteworthy that not only BMS, but other orofacial pain conditions are more prevalent in elderly patients, who generally have comorbid diseases in addition to physiological differences in the metabolism and excretion of drugs. Thus, studies that aim to assess the efficacy and safety of drugs for orofacial pain control in this population are clearly warranted. Interestingly, there are also many reports showing higher prevalence of many orofacial pain conditions in female subjects (4, 11), but the underlying mechanisms for the sex differences have just recently begun to be investigated.

In conclusion, this Research Topic gathered preclinical and clinical studies that presented perspectives and new therapeutic options for the treatment of different orofacial pain conditions. Collectively the studies reflect the diversity and complexity of orofacial pain physiopathology and management, and individually, each one added to the current literature valuable data toward a better understanding and management of chronic orofacial pain.
